# Research on factors affecting people’s intention to use digital currency: Empirical evidence from China

**DOI:** 10.3389/fpsyg.2022.928735

**Published:** 2022-08-05

**Authors:** Guo Wu, Jiangqin Yang, Qiaoxi Hu

**Affiliations:** Department of Finance, Shengxiang Business School, Sanda University, Shanghai, China

**Keywords:** digital currency, intention to use, financial knowledge, perceived value, exploratory factor analysis, confirmatory factor analysis, mediation analysis, structural equation modeling

## Abstract

In the era of FinTech, many countries are currently exploring the viability of their own digital currencies due to the vast potential in terms of efficiency, security and accessibility. Some digital currencies have been under rapid development and real-world trials have recently been deployed. The purpose of this paper is to understand the main factors that could affect people’s intention to use digital currency via an empirical study. A survey was employed to collect data and the final sample consisted of 408 respondents in China. The responses were analyzed using exploratory factor analysis, confirmatory factor analysis and structural equation modeling. The results showed that financial knowledge, perceived value, openness to innovation and perceived convenience positively impact people’s intention to use digital currency. It was also found that perceived value can be significantly anteceded by perceived monetary value, perceived functional value, and perceived emotional value. In addition, the mediating effect of perceived value on the influencing path between financial knowledge and intention to use was also confirmed. The findings can be utilized by governmental related authorities or FinTech companies to enhance the perception of users and develop effective strategies for increasing their intention to use digital currency.

## Introduction

Digital currency (DC) is any currency that is used exclusively in electronic form and not in physical form (Chuen, 2015). The rapid growth of internet usage and online purchasing habits have led to the creation of digital currency, which has all intrinsic properties of physical currency and allows for instantaneous transactions that can be easily executed across borders via supported devices and networks (Matsuura, 2016; Lee et al., 2021). Digital currency is often regarded as an innovative medium of exchange and it transforms the way transactions are conducted. But unlike physical currency, digital currency can only be electronically created and stored on computer systems or mobile devices (Chuen, 2015; Yao, 2018).

A central bank digital currency (CBDC) is a digital currency that can solely be issued and backed by a country’s central authority. As a legal tender, a CBDC must be accepted by all economic actors for any legal purposes, such as paying utility bills and paying taxes. Following the launch of decentralized cryptocurrencies such as Bitcoin and Ethereum, which are currently accounted for as indefinite-lived intangible assets (Tan and Low, 2017), central banks and governments around the world are exploring the possibility of establishing their own digital currencies (Bindseil, 2019; Lee et al., 2021). For example, the possibility of launching an American CBDC is currently under evaluation in Project Hamilton initiated by the Federal Reserve. The central bank of UAE is also working on a project to create its own CBDC. China launched the digital yuan project in 2014 and it is currently in the phase of testing functionality at provincial scale. The digital yuan has all the functions of China’s fiat currency and it is officially regulated as M0 money (Qian, 2019; Peters et al., 2020; Lu and Chen, 2021). Unlike decentralized cryptocurrencies, China’s digital yuan is issued and managed by China’s central bank using a centralized approach. China’s central bank is also responsible for unifying application standards, technical specifications and safety standards (Yao, 2018; Qian, 2019).

There are many advantages of using digital currency. First, the use of digital currency would help reduce environmental pollution (Wadsworth, 2018) and the transactions can go completely paperless. Second, the use of digital currency can help increase the efficiency of transactions (Lee et al., 2021). Transactions via digital currencies can be completed at any time, even during weekends when banks are closed. Faster processing time and enhanced security are key features (Smith and Weismann, 2014). It is expected that digital currency will become the main mode of payment in most countries. Its combination of security and convenience will make it a better alternative to the traditional money system (Yao, 2018). Third, the use of credit cards may run the risk of leaking personal information and this could cause fraudulent activities, while digital currency cannot be counterfeited and the transaction cannot be reversed arbitrarily (Chuen, 2015; Qian, 2019). However, there are some disadvantages that have been discussed by researchers. For example, some researchers (Gilbert and Loi, 2018; Latimer and Duffy, 2019; Samudrala and Yerchuru, 2021) have pointed out that using digital currency requires a user’s knowledge to properly perform certain tasks, such as opening digital wallets, making payments, and exchanging different digital currencies. In addition, some researchers (Gilbert and Loi, 2018; Lee et al., 2021) argued that there are still security issues associated with digital currencies as hackers could hack into devices and steal the private key of a digital wallet.

Digital payment tools like Alibaba’s Alipay and Tencent’s WeChat, which mainly rely on QR codes to complete transactions, have now been widely used by small businesses and individuals (Lu, 2018). The Chinese central government has therefore been motivated to expand the China digital yuan project and many hi-tech companies are also making investment to harness the promising business opportunities offered by the digital currency (Qian, 2019), which is officially called the Digital Currency Electronic Payment (DC/EP). Unlike blockchain-based cryptocurrencies, the DC/EP is centralized and not anonymous. It is expected that the DC/EP could help China accelerate the move to a cashless society and bring unbanked population into the economy. Some experts (Yao, 2018; Li, 2019; Shen and Hou, 2021) also state that the use of the DC/EP system could help detect money laundering transactions and track suspicious financial activities.

Prospects of the digital currency seem to be quite encouraging, but there is still a long way to go to achieve mass adoption by the general public in China. Much uncertainty still exists about people’s intention to use digital currency in spite of the availability. However, very little currently is known about the factors that predict people’s behavioral intentions in a digital currency context. We therefore undertake an analysis of factors affecting people’s intention to use digital currency by using empirical evidence from China. A conceptual model was established based on the modification of the unified theory of acceptance and use technology (UTAUT) model and four influencing constructs are proposed to affect the behavioral intention to use digital currency, together with the incorporation of a novel perceived value framework consisting of multidimensional constructs. A structural equation modeling approach is utilized in this study. It is expected that the current study can make a contribution to the existing literature by offering some important insights on factors affecting intention to use digital currency, providing policy implications and shedding light on effective strategies for the promotion of digital currency.

The paper is structured as follows. The first section of this paper will discuss the current contextual environment in China with respect to the implementation of digital currencies, as it could greatly impinge on the usage of digital currencies. Policy development, planning and decision-making also depend on the understanding of macro contextual factors. The next section will lay out the conceptual framework of the structural model and illustrate proposed factors affecting the intention to use of digital currency. The next section will then focus on various aspects of research methodology employed in the study. The findings of the research will then be presented and discussed, including results of validity analysis, reliability analysis, path coefficient analysis and mediation analysis. The policy implications of research findings will then be discussed. Based on the present study, the implications for promoting or marketing the use of digital currency are also shown.

## Research background

China has been researching digital fiat currency since 2014 and the People’s Bank of China (PBOC) established the Digital Currency Institute (DCI) and proposed the first prototype of China’s CBDC in 2016. In 2017, the PBOC started to develop and test the digital currency framework through cooperation with commercial banks, high-tech companies and telecom operators. So far CBDC pilots have been launched in Shenzhen, Shanghai, Suzhou, Chengdu and other cities where the digital economy is rapidly growing. The CBDC has also been featured at the 2022 Winter Olympics in Beijing. From these initiatives, it can be seen that China is serious about the digital currency project.

The economic environment in China calls for a new retail payment infrastructure that can be in line with the booming digital economy. The Chinese government has shifted their focus to high-quality economic growth (Li and Yang, 2020; Zakic, 2021) and the digital economy will be an important driver. A more inclusive and safer retail payment infrastructure, which meets diversified payment needs with enhanced convenience and security, is therefore needed to support the development of the digital economy. Meanwhile, the rapid development of mobile payment in China has helped the public get used to cashless payment. Using Alipay or WeChat pay in daily transactions has already become a habit for almost everyone in China (Lu, 2018). A survey conducted by the PBOC in 2019 has indicated that the number of transactions via mobile payment accounted for 66 percent, while cash payment only accounted for 23 percent (Li and Huang, 2021). The current social environment may help foster people’s positive beliefs and attitudes toward digital currencies that can further make sense of their financial lives.

The institutional environment in China is currently in neutrality but with increasing recognition of the need for relevant institutional arrangements and rules. On one hand, the PBOC has been quite prudent since the launch of research and development of the CBDC and there is still no special regulation in China. Some experts hold the view that the CBDC could cause financial disintermediation and weaken the efficacy of monetary policy (Bindseil, 2019; Shen and Hou, 2021). The PBOC has also claimed that close attention would be paid to the potential negative impacts on monetary system, financial markets and financial stability. On the other hand, as mentioned in China’s 14th 5-Year plan (Li and Yang, 2020; Poo, 2021), the Chinese government will forge ahead with the revisions of laws and regulations, such as the Law on the People’s Bank of China, regulations on personal information protection, etc. This could become a catalyst for the implementation of digital currencies in China. Overall, China is following the principle of being steady, managed and practical.

The present study offers some important insights into the development and usage of digital currencies in China. First, it helps in identifying decisive factors affecting the usage and the PBOC could accordingly improve the digital currency design or features and shape the business framework that can better suit the interests of the general public. Second, it provides policy makers and regulators with an in-depth understanding of user perceptions and helps them to assess how to promote the public acceptance of digital currency innovation while safeguarding financial stability and customer protection with appropriate regulatory arrangements and rules.

## The conceptual model

### Intention to use

The intention to use (IU) reflects the extent of an individual’s tendency to engage in a certain behavior (Davis et al., 1989; Cooper and Zmud, 1990). The theory of reasoned action (TRA) was first proposed by Fishbein and Ajzen (1980) and the model describes that people’s intention to use is impacted by people’s beliefs and attitudes. Based on the TRA, Ajzen (1991) further proposed the theory of planned behavior (TPB) to address the positive relationship between behavioral intention and behavioral action. The technology acceptance model (TAM) was then proposed by Davis (1989) and it shows the interaction between beliefs, attitudes and intention to use technological products (Venkatesh, 1999). A variety of researchers (Straub et al., 1997; Venkatesh and Davis, 2000; Deng et al., 2005; Teo, 2006; Ngai et al., 2007; Chow et al., 2012; Arias-Oliva et al., 2019) have adopted the TAM to conduct their specific theoretical and empirical studies and many results were in favor of the TAM (Venkatesh, 1999; Lee et al., 2003; Legris et al., 2003). Venkatesh et al. (2003) proposed the UTAUT model, which consists of four constructs (performance expectancy, effort expectancy, social influence and facilitating conditions) influencing intention and usage of information technology. To improve explanatory power, Venkatesh et al. (2012) further incorporated three constructs (hedonic motivation, price value, and habit) into the original UTAUT model to formulate the UTAUT2 model. Williams et al. (2015) reviewed the literature on the UTAUT and the UTAUT2 models and found that mixed results of factors predicting behavioral intentions were reported by researchers, in terms of constructs’ influencing strengths, statistical significance and corresponding items, while most previous studies were consistent with the postulations stated by Venkatesh et al. (2003). However, Dwivedi et al. (2019) argued that the original UTAUT model does not include any individual characteristic like attitude and self-efficacy toward behavioral intention. In the field of financial technology (FinTech), some researchers have extended either the UTAUT model or the UTAUT 2 model to study the intention to use FinTech products or services. For example, Zhou et al. (2010) adapted the UTAUT model to study factors affecting the user adoption of mobile banking and they found that the user adoption was significantly affected by task technology fit, performance expectancy, social influence and facilitating conditions. Similar results were reported by Bhatiasevi (2016). Kwateng et al. (2019) applied the UTAUT2 model to examine factors that influence the user adoption of mobile banking in Ghana and their findings suggest that price value, trust and habit are the main factors. Khatimah and Halim (2014) employed the UTAUT model to study the customer intention to use e-money in Indonesia, while Novendra and Gunawan (2017) studied the intention to use Bitcoin based on some UTAUT variables.

From the above discussion, it can be seen that the classic UTAUT model has gained popularity among researchers as it at least provides a reliable fundamental framework to start with and becomes the prism through which to analyze behavioral intention to use technological products. To effectively evaluate determinants predicting the intention to use digital currency, we propose a revised model by modifying or replacing constructs of the original UTAUT model with respect to the context of our study. The rationales for including these constructs in the conceptual model will be further specifically discussed in the following sections.

### Financial knowledge

Financial knowledge (FK) can be defined as the degree of knowledge that individuals have about various financial concepts (Stolper and Walter, 2017) and it would help people make informed financial decisions. Some researchers have demonstrated that financial knowledge is a predictive factor of behavioral intention to use financial products and services. Lusardi and Mitchell (2014) found that a better command of financial knowledge, a person’s higher intention to participate in financial markets. Kumar and Karlina (2020) found that there was a positive influence of financial knowledge on college students’ intention to use credit cards in Greater Jakarta with a total of 302 valid samples. Similar results were reported by Hastings et al. (2012) and the findings suggest that financial knowledge positively affects financial decisions related to the use of credit cards and investment instruments.

In fact, the influence of knowledge on behavioral intentions may be traced back to the TPB proposed by Ajzen (1991). The theory provides a model which shows that the behavioral intentions toward adoption new technology can be predicted by beliefs through perceived behavioral control. Polonsky et al. (2013) has shown that the predictive power of the TPB can be enhanced by the incorporation of the knowledge variable and several studies (Zhang et al., 2015; Parash et al., 2020) have revealed the significant nexus between knowledge, beliefs and perceived behavioral control. Some consumer research studies (Hölscher and Strube, 2000; Karimi et al., 2015) have also shown that knowledge of products would significantly affect online purchasing behaviors. Akhtar and Das (2019) employed an extended TPB model to examine factors affecting an individual’s intention to use investment vehicles and the findings also suggest that financial knowledge would positively affect intention. Interestingly, some neuroscientists (Hansen et al., 2012) found that prior knowledge could shape neural networks and impact cortical activity, resulting in biased perceptions and decisions when compared to scenarios without prior knowledge. From the above analysis, it is indicated that knowledge can be a significant predictor of behavioral intention, we therefore add the variable financial knowledge to our research model and the following hypothesis is therefore proposed:

H1. Financial knowledge positively influences intention to use digital currency.

Meanwhile, there may exist an indirect influencing path between financial knowledge and intention to use. Some researchers (Akhtar and Das, 2019; Albaity and Rahman, 2019) found that there was a partial mediation relationship between financial knowledge and intention with the attitude being the mediator. Similar results have been previously reported by Lim et al. (2018), the authors studied the effect of financial knowledge on financial behavioral intention and found that risk perception was a mediator. The mediating role of perceived value has been reported in other various contexts (Kwon et al., 2007; Shafiq et al., 2011; Chen and Lin, 2019; Gao et al., 2021). Perceived value is a measure of overall value assessed individually and it is usually characterized as the trade between advantages and expenses brought about (Woodruff, 1997; Petrick, 2002; Roig et al., 2006). Some earlier studies (Zeithaml, 1988; Dodds et al., 1991) indicated that perceived value can be a powerful predictor of customers’ behavioral intention. Expected utility theory (Morgenstern and Von Neumann, 1953; Schoemaker, 1982; Frisch and Clemen, 1994) suggests that value judgments would impact intentions. If an individual evaluates a product to be high in value, this judgment reflects a more positive attitude. The individual is more inclined to possess a higher level of purchase intention (Chang and Wildt, 1994).

According to prospect theory proposed by Kahneman and Tversky (1979), value can be gauged by the amount of expected gains and losses. The value function in prospect theory suggests that rational decision makers would undervalue uncertain outcomes. Similarly, ambiguity aversion theory (Ellsberg, 1961) posits that decision makers, such as people deciding whether to use a new technology or whether to purchase a product, prefer a known risk to an ambiguous risk. Ambiguity can be alleviated through the addition of relevant information (Puto, 1987) and the awareness of more specific information means a higher level of product knowledge (Brucks, 1985; Blair and Innis, 1996). Some studies (Rao and Monroe, 1988; Cacciolatti et al., 2015) have indicated that the linkage between knowledge and perceived value can be significant. It is implied that an individual with more knowledge of a product tends to feel less uncertain about usage outcomes and evoke perceptions of higher value. Based on the above analysis, we suppose that there exists a mediation of perceived value between financial knowledge and intention to use digital currency. The following hypothesis is proposed:

H2a. Financial knowledge positively influences perceived value.

### Perceived value

Perceived value (PV) refers to the concept that describes individuals’ assessment of the merits of a product or service and the evaluation of its ability to meet their demands when other similar products or services are available (Petrick, 2002, 2004; Boksberger and Melsen, 2011). The concept perceived value is actually rooted in equity theory (Carrell and Dittrich, 1978) and it represents customers’ evaluation of the desired reward for the perceived costs that have been sacrificed (Bolton and Lemon, 1999). Perceived value can also be viewed as a tradeoff between perceived benefit and perceived cost or risk (Roig et al., 2006; Kim et al., 2007; Prodanova et al., 2019; Barbu et al., 2021; Hasan et al., 2021). Studies on perceived value have been carried out in various sectors, such as hospitality and tourism industry (Al-Sabbahy et al., 2004; Sanchez et al., 2006), service industry (Boksberger and Melsen, 2011), software industry (Helander and Ulkuniemi, 2012), automobile industry (Lee et al., 2017), etc. Sánchez-Fernández and Iniesta-Bonillo (2007) and Coutelle-Brillet et al. (2014) reviewed the literature regarding the perceived value construct. With respect to the relationship between perceived value and behavioral intention to use FinTech products or services, a number of studies have been reported. Xie et al. (2021) studied factors that affected peoples’ intention to use FinTech services like internet wealth management platforms and the findings suggest that perceived value is strongly related to people’s FinTech adoption intention. Hasan et al. (2022) empirically investigated the factors that impact university students’ intention to use cryptocurrency and found that perceived value was a significant factor. Similar finding was reported by Pakrou and Amir (2016) in the case of Bitcoin. Karjaluoto et al. (2019) discovered that perceived value has strong effects on usage intention of mobile payment services and the overall satisfaction. Based on cost-benefit theory, Lin et al. (2020) analyzed the effect perceived value on intention to use mobile payment services and the study also revealed a significant positive relationship.

In fact, perceived value is a construct that has its foundations in various theories, such as social exchange theory (Cropanzano and Mitchell, 2005), utility theory (Fishburn, 1968), labor theory of value (Cohen, 1979). Researchers have brought constructs with some level of relevance to the concept of perceived value in the theories and models of technology acceptance, such as perceived utility and perceived of ease of use (Davis, 1989) in TAM, relative advantage (Rogers, 1995) in IDT (innovation diffusion theory), performance expectance and effort expectance (Venkatesh et al., 2003) in UTAUT, pleasure (Kulviwat et al., 2007) in CAT (consumer acceptance theory). In 2007, Kim et al. (2007) firstly brought the construct perceived value to a value-based adoption model (VAM) as a critical predictor of the intention to adopt technology. The study by Kim et al. (2007) has shown that the adoption of technology by customers can be better explained in VAM than other traditional models.

From the above discussion, it can be seen that perceived value could significantly affect the intention to use digital currency. The following hypotheses are therefore proposed:

H2b. Perceived value positively influences intention to use digital currency.

Meanwhile, there is a sustained interest in investigating the antecedents that determine perceived value and purchase intention among researchers over the years (Bolton and Drew, 1991; Vieira, 2013). Scholars focused on construct definition and the components of value (Rossiter, 2002; Petrick, 2004; Boksberger and Melsen, 2011) as understanding customers’ value perceptions is essential for effective marketing. For example, there is a growing body of literature on value dimensions in retail settings. Followed the suggestion by Zeithaml (1988), early studies often used benefits and costs as antecedents of value (Sirohi et al., 1998; Dabholkar et al., 2000; Swait and Sweeney, 2000). Researchers then expanded studies on other components, such as perceived risk (Adapa et al., 2020), quality (Sweeney and Soutar, 2001), trust (Ponte et al., 2015), etc. Some researchers argued that emphasis should be placed on performance, quality, price, cost, risk, time and other economic utility related components (Petrick, 2002; Roig et al., 2006; Boksberger and Melsen, 2011). These notions are based on a rational perspective, suggesting that perceived functional value (PFV) (e.g., quality and performance) and perceived monetary value (PMV) (e.g., price and cost) can be established as the main contributors of perceived value. Therefore, we propose the following hypotheses:

H3a. Perceived monetary value positively predicts perceived value.

H3b. Perceived functional value positively predicts perceived value.

In recent years, the emotional component of perceived value has also received a great deal of attention (Sanchez et al., 2006; Lee et al., 2007; Khan and Mohsin, 2017; Kato, 2021). Emotional value can be defined as the reward derived from positive feelings and the states of pleasure and enjoyment when using a product or service (Sweeney and Soutar, 2001; Pura, 2005; Sanchez et al., 2006). Studies (Asshidin et al., 2016; Candi and Kahn, 2016) have shown that emotional value would be enhanced by experiencing something new or different. Dodds et al. (1991) suggested a positive linkage between emotional value and intention to purchase retail products, as customers tend to associate brands with specific symbolic meanings, which could enhance their feelings of joy and contentment. In fact, how customers view products emotionally may be traced back to motivation research (Dichter, 1960) which promotes the notion that non-cognitive and unconscious motives drive customers’ choices. Since the 1970s, various emotion theories then emerged to investigate connections between emotions and actions (Tomkins, 1970; Ekman and Friesen, 1971; Strongman, 1996; Huang, 2001; Plutchik, 2001; Izard, 2009). Some researchers have also argued that emotions should include cognitive content and there are rationality related features associated with emotions (Helm, 1994; Mulligan, 1998; Gabbay et al., 2006). But how emotional feelings or affective states could impact behavioral intentions is still unclear so far. As little research has been done to investigate whether perceived emotional value (PEV) can play a significant role in predicting perceived value, especially in a FinTech context. Based on the above analysis, we therefore incorporate a novel multidimensional perceived value framework, which consists of PFV, PMV, and PEV, into our theoretical model. This investigation is expected to provide insights on how perceived value is formed through various antecedent variables as well as the levels of influencing strength among relationships. The following hypotheses are proposed:

H3c. Perceived emotional value positively predicts perceived value.

In addition, it should be noted that the relationship between perceived value and intention to use digital currency in China may be dependent on its culture and contextual environment. Fan et al. (2018) performed a comparative study between China and United States on users’ attitude toward mobile payment service. The authors found that Chinese people tend to generate higher trust in mobile payment than Americans with the same level of mobile payment prevalence. This could be due to the fact that China has a more collectivistic culture (Hofstede, 2004, 2007; Ghosh, 2011) with stronger regulations being reinforced in the financial services sector (Huang and Wang, 2017). In China, people tend to value interdependence, harmony, and conformity (Hofstede, 2004). This implies that Chinese people may have more confidence in their perceived value of the digital currency issued by the PBOC. Similar findings have been noticed in other contexts. Hernandez-Ortega et al. (2017) investigated the effect of cultures with different degrees of collectivism on satisfaction of advanced mobile messaging services and found that the influence of perceived value on satisfactory is higher in Greece than in Spain. Liu et al. (2019) found that the effects of perceived value on mobile advertising acceptance are significantly different across countries with different cultural environments. Ashraf et al. (2021) also reported that there are country-specific levels in the impact of perceived value on customers’ usage motivation in a mobile commerce context.

### Openness to innovation

In the present study, openness to innovation (OI) refers to the concept which describes the degree of individuals’ innovativeness and it measures their personality openness and willingness to try innovative technological products or services. Theoretically, the effect of openness to innovation on behaviors can be traced back to Hofstede’s theory (De Mooij and Hofstede, 2010; Hofstede, 2011). The theory suggests an uncertainty avoidance dimension which reflects a country’s level of tolerance for uncertainty and unstructured situations. The willingness to embrace innovative products with uncertain prospects in a society can be influential in many aspects, such as customer behaviors, social norms and business strategy development. In the case of China, studies (Hofstede, 2004, 2007; Hofstede and Minkov, 2010) have shown that the level of uncertainty avoidance is relatively higher than countries such as United States and Australia. Davison and Jordan (1998) reckoned that technologies that facilitate the dissemination of information would be implemented in a country with a high uncertainty avoidance characteristic. This implies that an individual from China may require more formal rules and documentation of a product prior to acceptance.

Empirically, there are some studies indicating a significant nexus between openness to innovation and behavioral intention. Lassar et al. (2005) studied the role of innovativeness in predicting people’s online banking adoption. Yoon and Steege (2013) discovered that people with higher preference for innovation tend to be more curious and participative to adopt any new technology. Khan et al. (2019) investigated the acceptance of electronic banking in Pakistan and the findings were similar. Based on innovation diffusion theory (Rogers, 1995, 2002; McGrath and Zell, 2001), Gupta and Arora (2017) put forward a behavioral reasoning theory to explain the positive relationship between personal innovativeness and behavioral intention to use mobile banking. Ho et al. (2020) provided further empirical evidence to support the positive relationship. Simarmata and Hia (2020) also concluded that an individual with higher innovative personality is more likely to accept new information technology products.

According to the above discussion, openness to innovation could be a significant predictor of peoples’ behaviors toward the usage of digital currencies, we therefore propose the following hypothesis:

H4. Openness to innovation positively influences intention to use digital currency.

### Perceived convenience

The concept of perceived convenience (PC) describes the degree of convenience associated with time, place, and the simplicity of execution when it comes to the usage of a certain technological product or service (Yoon and Kim, 2007; Farquhar and Rowley, 2009; Chang et al., 2013; Teo et al., 2015). A number of researchers have adapted classic models via incorporating perceived convenience as one of the additional constructs. For example, Hsu and Chang (2013) built an extended TAM model by incorporating the perceived convenience variable which significantly predicts students’ intention to use and acceptance of an e-learning system. Bhatiasevi (2016) established an extended UTAUT model to explain the adoption of mobile banking by including perceived convenience as an influential variable. The adoption of FinTech products or services has been an interesting topic in recent years. Kim et al. (2015) illustrated that perceived convenience was one of the most influential variables on the usage intention of payment related FinTech services. Podile and Rajesh (2017) found that respondents’ perception about convenience had a positive impact on intention to adopt cashless transactions in India. Gao and Waechter (2017) also identified the positive relationship between perceived convenience and usage intention of mobile payment by analyzing a sample of 851 respondents in Australia. Pal et al. (2021) demonstrated that perceived convenience has a positive impact on individuals’ intention to use mobile payments via survey responses from a sample of 215 people. Digital currencies can provide convenience to the users in several ways. First, the digital currency operational system is adaptive to offline payments. The settlement of a transaction via mobile payment providers usually requires telecommunications or internet coverage. Digital currencies can serve the transaction needs in offline situations and this would help ensure business continuity. Second, the use of digital currencies is convenient and safe without linking any bank accounts. Individuals use personal digital wallets which enable them to make immediate payments, transfer funds, set payment conditions and caps, while enterprises use corporate digital wallets to pool or distribute funds. In addition, the digital currency in China is designed to be inclusive (Yao, 2018) and it must be accepted in all kinds of payment scenarios. This could eliminate the inconvenience caused by switching among different mobile payment apps.

As can be seen from the above discussion, perceived convenience could significantly affect intention to use digital currency, we therefore incorporate the variable into our conceptual model and propose the following hypothesis:

H5. Perceived convenience positively influences intention to use digital currency.

**FIGURE 1:**
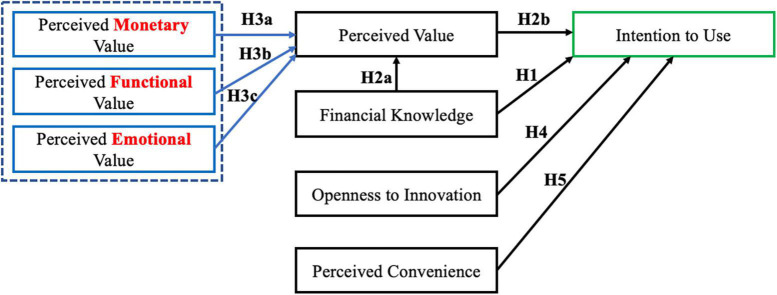
The hypothesized conceptual framework.

Overall, Figure 1 shows the conceptual model based on the hypotheses proposed for the relationships between the interested variables.

## Research methodology

### Sampling procedure

A survey was used to collect the quantitative data required for the study. We employed a combined sampling approach which includes random selection of online public chat groups and convenience sampling. The study targeted a population that consists of local Chinese residents aged above 18 years with basic experience in the use of the Internet and mobile phones. They are more capable of comprehending survey questions and rating things from their own perspectives. The groups were randomly selected from several lists of public chat groups and the conveniently available participants were chosen in order to achieve the desired sample size with ease (Etikan et al., 2016). The sampling procedure employed in the study is consistent with research methods adopted in other empirical studies of the intention to use FinTech products (Lim et al., 2019; Singh et al., 2021). Specifically, the invitation to respond to the survey and the associated link was distributed randomly online without considering respondents’ prior knowledge of FinTech products. The process regarding online data collection and storage was ensured to fulfill the guiding ethical principles. The study was performed in line with Sanda University’s policy on research with minimal risk involving human participants with the approval number of 2022003. The conveniently available participates were informed that the survey was for academic purpose only and all data would not be used for commercial purposes. The participation was strictly voluntary, and respondents were asked to read the ethical statement on the top of the questionnaire. They were also informed that they had the right to withdraw at any time during the study. The anonymity of the questionnaire and respondents’ confidentiality were both assured.

With respect to the desired sample size, researchers have suggested a variety of rules or guidelines when determining the sample size in empirical studies (Memon et al., 2020). For example, the well-known Krejcie and Morgan table (KMT) suggests that a sample size of 384 is enough for a population of one million or more (Krejcie and Morgan, 1970). Roscoe and Byars (1971) reckoned that a sample size between 30 and 500 is suitable for most behavioral studies. Gorsuch (1990) suggested that the sample-to-item ratio should be no less than 5:1 and a study of 25 items would require at least 125 respondents. Kline (2016) suggested that a sample size over 200 should be considered for analyzing structural equation models. A similar recommendation was put forward by Kock and Hadaya (2018). Based on the suggestions from the extant literature, a sample size of around 400 deems appropriate in the context of the present study.

### Survey design and data collection

In this study, the instrument for validating the conceptual model was a self-report survey questionnaire which served as the tool for collecting the research data. Each factor was measured through five indicators. All items used Likert-type scales with a graduation from 1 (minimum score, strongly disagree) to 5 (maximum score, strongly agree). All questions correspond to the conceptual model defined in the study and the wordings were carefully refined according to feedback from various experts in similar fields. A Chinese version was also established by translating the original English version via the back-translation technique (Brislin, 1970) to obtain clarity and meaning similarity. An initial pilot study was also conducted with 50 bilingual participants to make sure that both the questions in several key constructs and the response formats were understood. A revised version of the questionnaire (Appendix Table A1) with the incorporation of questions regarding the perceived value framework, was then reviewed and swiftly pretested by experts and professionals in Shanghai. After excluding incomplete responses, a final sample size of 408 respondents was obtained.

### Data analysis

The collected data was analyzed through a series of steps. First, the reliability of the questionnaire was assessed using the Cronbach’s alpha method (Tavakol and Dennick, 2011). Second, exploratory factor analysis (EFA) was performed using SPSS 26 to confirm the anticipated five factors and to test the convergent validity and discriminant validity. Third, confirmatory factor analysis (CFA) was performed with AMOS 26 to validate the measurement model by calculating composite reliability, standardized factor loadings, average variance extracted (AVE), and goodness-of-fit indices. Fourth, the hypothesized relationships between variables were tested by examining the structural equation model and the mediation analysis was conducted using maximum likelihood estimates and bootstrap resampling.

In hypothesis testing, both the significance of individual paths and the model as a whole were considered. Specifically, the significance of an individual path was evaluated by the *t* statistic which is equal to the ratio of path coefficient estimate to its standard error (known as the critical ratio, CR). It is noted that as the sample size increases, the CR could resemble a normal distribution (Gao et al., 2008), implying that a value of CR greater than 1.96 means the path is significant at the 5% level. Meanwhile, as for the evaluation of the model as a whole, various test statistics had been recommended. According to Bentler and Bonett (1980), the Chi-square χ^2^ test statistic assesses the fit between the hypothesized model and the observed results. Barrett (2007) pointed out that the Chi-square χ^2^ test statistic is more reliable than other model fit indices, some scholars (Singh, 2009; Kline, 2016; Hair et al., 2018) also recommended the use of the Chi-square χ^2^ test statistic divided by the degree of freedom (*df*) as a measure, with a value of 3 or less being a common cutoff (Hu and Bentler, 1999). However, some scholars (Marsh et al., 1988; Kenny and McCoach, 2003; Schumacker and Lomax, 2010) criticized that the Chi-square χ^2^ test statistic may always be significant when the sample size is large. The goodness-of-fit index (GFI) and the adjusted goodness-of-fit index (AGFI) have also been used extensively in the literature (Joreskog and Sorbom, 1982; Tanaka and Huba, 1985; Lei and Lomax, 2005). Sharma et al. (2005) criticized these measures for being sensitive to sample size. Other alternative measures are recommended, such as incremental fit index (IFI) (Bollen, 1989) and normed fit index (NFI) (Bentler, 1990). The Tucker-Lewis index (TLI) (Tucker and Lewis, 1973; Hu and Bentler, 1999; Kenny and McCoach, 2003) and the comparative fit index (CFI) (Fan et al., 1999) are also preferred as they provide some penalties for adding parameters. Another popular statistic is the root mean square error of approximation (RMSEA) as confidence intervals of the point estimate can be computed (Chen et al., 2008). MacCallum et al. (1996) suggested a cutoff value of 0.08 and RMSEA may produce better rejection rates, while Kenny et al. (2015) argued that models with small *df* and small sample size could produce artificially large values. Overall, there are still some debates among scholars on which test statistic should be preferred, implying that it is unlikely to find a single test statistic that can both penalize model complexity and maintain stable across different sample sizes and distributions. Therefore, a set of test statistics will be reported so as to assess the consistency of model fitting results.

### Profile of respondents

The final respondents include 408 local Chinese residents and their profiles are summarized as follows: 53% of the respondents were male, while 47% were female; the age levels of the respondents were in the range of 18–30 (54%), 31–55 (41%), and 56–65 (5%) respectively; the income levels of the respondents were in the range of below 50000 CNY per year (27%), 50000–100000 CNY per year (55%), and above 100000 CNY per year (18%), respectively; 32% of the respondents were living in rural areas, while 68% were living in urban areas.

## Results and discussion

### Reliability test

The internal consistency of the questionnaire was measured by calculating the Cronbach’s alpha (α) of each multi-item scale. The value of Cronbach’s alpha ranges from 0 to 1. A higher Cronbach’s alpha indicates more shared covariances between scale items which reliably measure the same variable (Cronbach, 1951; Cortina, 1993). Some researchers agreed (Tavakol and Dennick, 2011; Bonett and Wright, 2015; Taber, 2018) that a value of α > 0.7 is generally acceptable for most research while other researchers (Cho and Kim, 2015; Heo et al., 2015) argued that more stringent cut-offs (e.g., α > 0.8) should be considered. As shown in Table 1, all the Cronbach’s alpha values were well above the recommended cut-off: PMV, α = 0.873; PFV, α = 0.862; PEV, α = 0.873; financial knowledge (FK), α = 0.861; openness to innovation (OI), α = 0.831; perceived convenience (PC), α = 0.867; perceived value (PV), α = 0.856; and intention to use (IU), α = 0.856. The corrected item-total correlation (CITC) values are also above 0.5, indicating the coherence between items and an appropriate discrimination in questions. Table 1 also presents the value of Cronbach’s alpha would be if a particular question was removed from the scale. It can be seen that the removal of any question in each scale would result in a lower Cronbach’s alpha, suggesting a high level of reliability between items.

**TABLE 1:** Results of the Cronbach’s alpha values.

Variable	Item	CITC	Cronbach’s alpha if item deleted	Cronbach’s alpha
Perceived monetary value (PMV)	PMV1	0.716	0.843	0.873
	PMV2	0.657	0.858	
	PMV3	0.705	0.845	
	PMV4	0.699	0.846	
	PMV5	0.739	0.837	
Perceived functional value (PFV)	PFV1	0.724	0.824	0.862
	PFV2	0.717	0.825	
	PFV3	0.747	0.817	
	PFV4	0.657	0.841	
	PFV5	0.577	0.860	
Perceived emotional value (PEV)	PEV1	0.727	0.840	0.873
	PEV2	0.710	0.844	
	PEV3	0.711	0.844	
	PEV4	0.677	0.852	
	PEV5	0.679	0.851	
Financial knowledge (FK)	FK1	0.719	0.823	0.861
	FK2	0.647	0.841	
	FK3	0.635	0.845	
	FK4	0.723	0.822	
	FK5	0.684	0.832	
Openness to innovation (OI)	OI1	0.563	0.816	0.831
	OI2	0.623	0.800	
	OI3	0.715	0.774	
	OI4	0.661	0.789	
	OI5	0.591	0.809	
Perceived convenience (PC)	PC1	0.756	0.822	0.867
	PC2	0.710	0.833	
	PC3	0.677	0.842	
	PC4	0.646	0.849	
	PC5	0.656	0.847	
Perceived value (PV)	PV1	0.698	0.821	0.856
	PV2	0.639	0.836	
	PV3	0.687	0.823	
	PV4	0.641	0.834	
	PV5	0.696	0.820	
Intention to use (IU)	IU1	0.743	0.811	0.856
	IU2	0.712	0.816	
	IU3	0.687	0.823	
	IU4	0.574	0.854	
	IU5	0.659	0.830	

### Validity test

#### Exploratory factor analysis

An initial EFA was conducted to examine the 40 scale items representing financial knowledge, openness to innovation, perceived convenience, perceived value and intention to use. EFA is a statistical method which can be used to help determine the number of latent constructs and identify the underlying factor structure (Fabrigar et al., 1999), especially when the information of dimensionality is limited. The analysis was carried out using VARIMAX rotation with SPSS 26 and the final eight-factor solution explained 65.4% of the variance (see Table 2A). The Kaiser-Meyer-Olkin (KMO) test returned a value of 0.932, indicating the sampling is adequate. The Bartlett’s test for sphericity was also conducted to ensure moderate intercorrelations between items and the test returned a value of 8306.447 (*p* = 0.000), suggesting the suitability for factor analysis. It can be seen from Table 2B that the EFA resulted in the anticipated five factors. As the items load more strongly on their associated factors (>0.50) than on the other factors, convergent validity and discriminant validity are both evident.

**TABLE 2A:** Results of the exploratory factor analysis (total variance explained).

Factor	Initial eigenvalues			Extraction SSL			Rotation SSL		
			
	Total	% of Var	CUM%	Total	% of Var	CUM%	Total	% of Var	CUM%
1	11.921	29.803	29.803	11.921	29.803	29.803	3.436	8.591	8.591
2	2.537	6.344	36.146	2.537	6.344	36.146	3.374	8.436	17.027
3	2.377	5.943	42.090	2.377	5.943	42.090	3.365	8.411	25.439
4	2.222	5.554	47.644	2.222	5.554	47.644	3.291	8.227	33.666
5	1.986	4.965	52.609	1.986	4.965	52.609	3.269	8.174	41.840
6	1.948	4.869	57.478	1.948	4.869	57.478	3.220	8.051	49.890
7	1.654	4.134	61.613	1.654	4.134	61.613	3.130	7.825	57.715
8	1.531	3.828	65.441	1.531	3.828	65.441	3.090	7.726	65.441

SSL, sums squares of loadings; CUM, cumulative; criterion: eigenvalue > 1; varimax rotation.

**TABLE 2B:** Results of the exploratory factor analysis (component matrix).

	Factors							
	
Items	1 (PMV)	2 (PEV)	3 (PC)	4 (FK)	5 (PV)	6 (IU)	7 (PFV)	8 (OI)
FK1	0.147	0.123	0.091	0.773	0.157	0.098	0.144	0.058
FK2	−0.001	0.127	0.131	0.743	0.117	0.099	0.106	0.093
FK3	0.109	0.132	0.036	0.688	0.072	0.167	0.219	0.097
FK4	0.040	0.055	0.113	0.790	0.103	0.188	0.096	0.122
FK5	0.168	0.080	0.062	0.698	0.226	0.190	0.202	0.072
OI1	0.143	0.136	0.074	0.039	0.078	0.066	0.074	0.684
OI2	0.020	0.060	0.142	0.083	0.012	0.075	0.206	0.739
OI3	0.145	0.055	0.119	0.105	0.084	0.107	0.135	0.787
OI4	0.094	0.123	0.122	0.045	0.117	0.178	0.134	0.728
OI5	0.115	0.065	0.079	0.158	0.220	0.114	0.081	0.668
PC1	0.092	0.135	0.796	0.075	0.176	0.102	0.075	0.176
PC2	0.074	0.089	0.777	0.088	0.113	0.116	0.128	0.119
PC3	0.121	0.103	0.752	0.086	0.022	0.135	0.089	0.110
PC4	0.175	0.130	0.727	0.055	0.032	0.142	0.103	0.023
PC5	0.066	0.128	0.748	0.107	0.079	0.059	0.077	0.117
PV1	0.111	0.142	0.136	0.151	0.743	0.115	0.121	0.105
PV2	0.103	0.193	0.075	0.209	0.686	0.134	0.067	0.095
PV3	0.159	0.153	0.090	0.082	0.747	0.194	0.118	0.024
PV4	0.110	0.110	0.095	0.079	0.707	0.139	0.145	0.124
PV5	0.067	0.068	0.036	0.135	0.772	0.154	0.100	0.163
IU1	0.154	0.095	0.111	0.168	0.205	0.758	0.123	0.131
IU2	0.086	0.136	0.113	0.215	0.127	0.753	0.135	0.115
IU3	0.135	0.098	0.155	0.138	0.129	0.741	0.116	0.096
IU4	0.065	0.040	0.105	0.075	0.158	0.668	0.136	0.100
IU5	0.099	0.139	0.108	0.152	0.129	0.717	0.101	0.130
PMV1	0.768	0.086	0.133	0.046	0.098	0.105	0.158	0.135
PMV2	0.710	0.098	0.179	0.155	0.072	0.119	0.105	0.112
PMV3	0.784	0.041	0.058	0.118	0.187	0.066	0.088	0.108
PMV4	0.752	0.136	0.087	0.086	0.087	0.080	0.185	0.136
PMV5	0.803	0.159	0.096	0.019	0.092	0.144	0.137	0.047
PFV1	0.217	0.196	0.123	0.193	0.162	0.135	0.685	0.219
PFV2	0.133	0.129	0.178	0.210	0.149	0.184	0.703	0.166
PFV3	0.078	0.155	0.095	0.141	0.116	0.133	0.808	0.114
PFV4	0.140	0.044	0.121	0.129	0.150	0.102	0.736	0.109
PFV5	0.227	0.033	0.055	0.162	0.051	0.129	0.630	0.162
PEV1	0.080	0.778	0.154	0.121	0.135	0.060	0.158	0.064
PEV2	0.074	0.768	0.219	0.037	0.083	0.075	0.182	0.074
PEV3	0.131	0.744	0.184	0.165	0.103	0.132	0.047	0.138
PEV4	0.109	0.760	0.026	0.127	0.116	0.095	0.111	0.101
PEV5	0.125	0.759	0.062	0.064	0.205	0.126	−0.026	0.091

#### Confirmatory factor analysis

**FIGURE 2:**
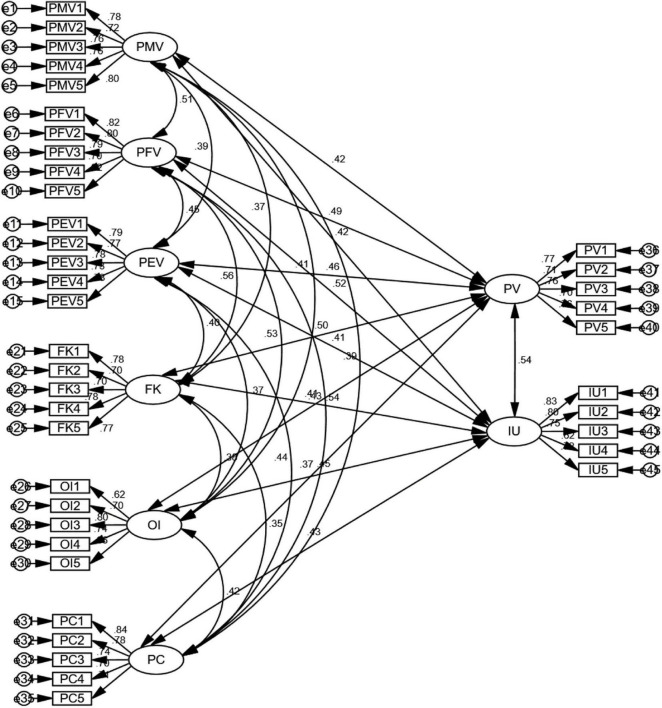
Diagram illustrating the measurement model.

To further assess the construct validity, CFA was performed using SPSS Amos 26. It is known that CFA can help test the consistence between observed data and the hypothesized conceptual model which specifies the assumed relations between latent factors (Brown, 2015). CFA also offers a measurement model based on structural equations modeling (SEM). The results are shown in Figure 2, Tables 3, 4. First of all, as can be seen in Table 3, the composite reliability ranges from 0.834 to 0.876, exceeding the acceptable level of 0.70 recommended by various researchers (Bacon et al., 1995; Shrestha, 2021). AVE scores for constructs are all above the acceptable variance-extracted level of 0.50 recommended by Hulland (1999) and Brown (2015), indicating convergent validity. Second, the standardized factor loadings of items range from 0.616 to 0.836 and they are all higher than the cutoff value of 0.50 (Brown, 2015). Third, as shown in Table 4, the GFIs of the measurement model are all acceptable: χ2/degree of freedom (CMIN/DF) = 1.084; GFI = 0.916; RMSEA = 0.024; NFI = 0.910; IFI = 0.992; TLI = 0.992; CFI = 0.992; Overall, the results of the CFA are satisfactory, supporting the subsequent structural model analysis.

**TABLE 3:** Results of the confirmatory factor analysis.

Factor	Items	Unstandardized loading	S.E.	Standardized loading	CR	AVE	*p*
PMV	PMV1	1.000		0.781	0.876	0.586	
	PMV2	1.118	0.076	0.718			***
	PMV3	1.134	0.072	0.762			***
	PMV4	1.103	0.070	0.761			***
	PMV5	1.062	0.064	0.802			***
PFV	PFV1	1.000		0.819	0.866	0.566	
	PFV2	1.069	0.060	0.805			***
	PFV3	1.009	0.058	0.792			***
	PFV4	0.996	0.066	0.704			***
	PFV5	0.839	0.064	0.624			***
PEV	PEV1	1.000		0.793	0.874	0.581	
	PEV2	1.025	0.063	0.775			***
	PEV3	0.972	0.059	0.784			***
	PEV4	0.967	0.064	0.728			***
	PEV5	0.940	0.062	0.728			***
FK	FK1	1.000		0.781	0.864	0.561	
	FK2	0.975	0.069	0.700			***
	FK3	0.974	0.069	0.699			***
	FK4	0.999	0.062	0.783			***
	FK5	0.940	0.059	0.775			***
OI	OI1	1.000		0.621	0.834	0.504	
	OI2	1.252	0.111	0.702			***
	OI3	1.338	0.109	0.803			***
	OI4	1.288	0.110	0.744			***
	OI5	1.171	0.109	0.663			***
PC	PC1	1.000		0.836	0.867	0.568	
	PC2	0.921	0.054	0.777			***
	PC3	0.878	0.055	0.737			***
	PC4	0.810	0.054	0.697			***
	PC5	0.843	0.055	0.713			***
PV	PV1	1.000		0.772	0.858	0.548	
	PV2	1.042	0.074	0.705			***
	PV3	1.129	0.074	0.763			***
	PV4	0.946	0.068	0.699			***
	PV5	1.090	0.072	0.758			***
IU	IU1	1.000		0.831	0.861	0.556	
	IU2	1.059	0.060	0.798			***
	IU3	1.023	0.063	0.747			***
	IU4	0.883	0.069	0.616			***
	IU5	0.924	0.060	0.718			***

***p < 0.001.

**TABLE 4:** Goodness-of-fit indices of the measurement model.

χ ^2^	CMIN/DF	GFI	RMSEA	NFI	IFI	TLI	CFI
	<3	>0.9	<0.08	>0.9	>0.9	>0.9	>0.9
772.058	1.084	0.916	0.024	0.910	0.992	0.992	0.992

#### Correlation analysis

Correlation analysis was also performed to assess discriminant validity, which represents the extent that measures of constructs minimally correlation with one another (Matthes and Ball, 2019). Specifically, the square root of the AVE for each factor was compared with the inter-construct correlation coefficients in the correlation matrix. As can be seen in Table 5, the inter-construct correlation coefficients are all below 0.5 and significantly lower than the square roots of the AVEs, indicating satisfactory divergence between different constructs. The results of correlation analysis also support a further structural model analysis.

**TABLE 5:** Discriminant validity for the measurement model (implied correlation matrix).

Factors	PMV	PFV	PEV	FK	OI	PC	PV	IU
PMV	0.766^a^							
PFV	0.510	0.752^a^						
PEV	0.391	0.447	0.762^a^					
FK	0.369	0.563	0.401	0.749^a^				
OI	0.406	0.527	0.368	0.382	0.710^a^			
PC	0.386	0.430	0.438	0.354	0.420	0.754^a^		
PV	0.418	0.490	0.464	0.496	0.413	0.374	0.740^a^	
IU	0.420	0.517	0.408	0.539	0.446	0.428	0.537	0.746^a^

^a^Square root of average variance extracted (AVE).

### Test of the structural model

#### Goodness of fit

**FIGURE 3:**
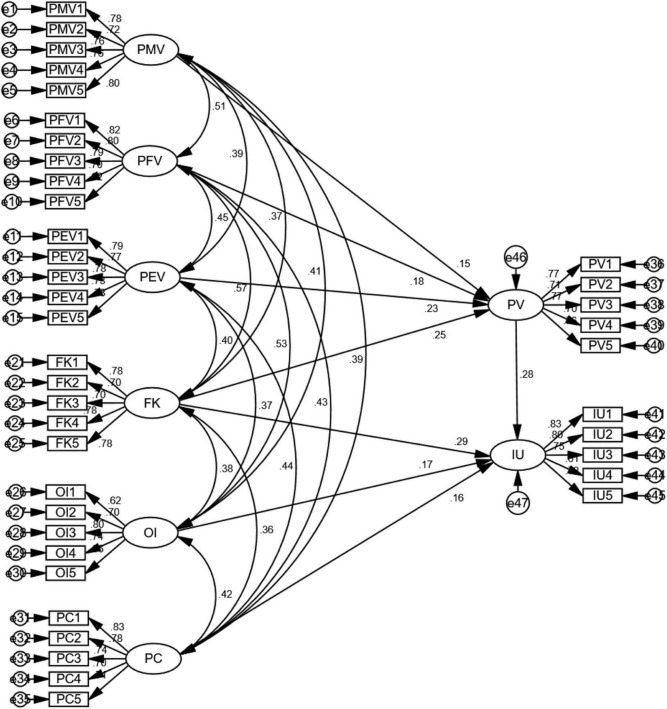
Diagram illustrating the structural model.

Structural equation modeling was adopted to test the relationships proposed in the hypothesized model, as shown in Figure 3. It can be seen in Table 6 that all goodness-of-fit indicators were found to exceed the levels recommended by the literature (Singh, 2009; Iacobucci, 2010; Hair et al., 2019): χ2/degree of freedom (CMIN/DF) = 1.092; GFI = 0.915; RMSEA = 0.024; NFI = 0.909; IFI = 0.992; TLI = 0.991; CFI = 0.992; The structural model is therefore deemed acceptable.

**TABLE 6:** Goodness-of-fit indices of the structural model.

χ ^2^	CMIN/DF	GFI	RMSEA	NFI	IFI	TLI	CFI
	<3	>0.9	<0.08	>0.9	>0.9	>0.9	>0.9
783.132	1.092	0.915	0.024	0.909	0.992	0.991	0.992

#### Path coefficient analysis

The path coefficient analysis (Land, 1969) was conducted with SPSS AMOS 26 to test the proposed causal relationships between constructs. Table 7 shows the standardized path coefficients of the structural model with the statistical significance. It is evident that the relations between construct were all positively significant. First, the positive relationship between financial knowledge and intention to use was demonstrated with a significant standardized coefficient (β = 0.288, *p* < 0.001). Financial knowledge was also found to be a significant and positive predictor of perceived value (β = 0.246, *p* < 0.001), whilst perceived value was also found to have a significant and positive relationship with intention to use (β = 0.277, *p* < 0.001). Second, PMV, PFV, and PEV can significantly predict perceived value, with the standardized coefficients of 0.152 (*p* < 0.05), 0.179 (*p* < 0.05), and 0.231 (*p* < 0.001), respectively. Third, openness to innovation was shown to have a significant and positive impact on intention to use (β = 0.166, *p* < 0.01). Fourth, perceived convenience was found to be a significant predictor of intention to use (β = 0.161, *p* < 0.01). Therefore, the results indicate that all hypotheses with the proposed causal relationships were supported at the *p* < 0.01 level.

**TABLE 7:** Results of the path coefficient analysis.

Hypotheses	Path coefficient (β)	S.E.	C.R. (*t*-value)	*p*	Decision
H1: FK → IU	0.288	0.061	4.816	***	Supported
H2a: FK → PV	0.246	0.062	3.836	***	Supported
H2b: PV → IU	0.277	0.062	4.754	***	Supported
H3a: PMV → PV	0.152	0.066	2.550	*	Supported
H3b: PFV → PV	0.179	0.068	2.585	*	Supported
H3c: PEV → PV	0.231	0.050	3.958	***	Supported
H4: OI → PV	0.166	0.066	2.932	**	Supported
H5: PC → PV	0.161	0.044	2.979	**	Supported

*p < 0.05; **p < 0.01; ***p < 0.001.

#### Mediation analysis

The mediation process was also tested using SEM in the present study. As suggested by Baron and Kenny (1986), SEM provides a more appropriate mediation analysis than the method of regressing equations. Specifically, the mediating effect of perceived value on a relationship between financial knowledge and intention to use was examined. The bootstrap method (Rucker et al., 2011; Gunzler et al., 2013) was employed in the mediation analysis as it does not rely on any distribution assumption of on the indirect effect. As can be seen in Table 8, the mediating effect of perceived value on the path between financial knowledge and intention to use is confirmed. We calculated both bias-corrected confidence intervals and percentile confidence intervals at the 95% significance level. The results show that the value of the indirect effect regarding the FK-PV-IU path is within the confidence intervals, suggesting that perceived value partially mediates the path between financial knowledge and intention to use.

**TABLE 8:** Results of the mediation analysis.

Paths	Indirect Effect (Standardized β)	Bias-Corrected 95% CI	Percentile 95% CI
PMV → PV → IU	0.042	0.004–0.105	0.003–0.098
PFV → PV → IU	0.050	0.007–0.117	0.005–0.112
PEV → PV → IU	0.064	0.019–0.144	0.013–0.132
FK → PV → IU	0.068	0.020–0.149	0.015–0.139

Bootstrap Reps = 2000; CI, confidence interval.

### Discussion

The present study was designed to analyze the factors impacting people’s intention to use digital currency. First of all, the findings suggest that financial knowledge is significantly related to intention to use. This is in line with most existing studies on behavioral intentions to use financial products and services (Luarn and Lin, 2005; Kang and Choi, 2019; Lim et al., 2019; Muslichah and Sanusi, 2020). A partial mediation effect was also confirmed as perceived value mediates the path between financial knowledge and intention to use. The results may corroborate the viewpoint that perceived value (in terms of functionality and utility) depends on the degree of knowledge that a person has acquired (Boksberger and Melsen, 2011). If people were unfamiliar with finance related concepts, they were unlikely to gauge the value of FinTech products accurately. Meanwhile, it was shown that openness to innovation had a positive impact on intention to use. The findings were consistent with similar empirical studies (Islam et al., 2013; Cheng, 2014; Hong et al., 2017). A possible explanation could be based on diffusion of innovation theory (Rogers, 1995, 2002). The theory states that innovators have more willingness to try new technological things than the majority and thus they have greater chances for adoption. However, it should also be noted that the theory assumes innovation is solely beneficial and is just waiting to be adopted. In addition, the findings further promote the notion that perceived convenience would positively influence intention to use. The results were in agreement with similar studies carried out in Malaysia (Lai and Liew, 2021), India (Pal et al., 2021), South Korea (Joo, 2018), and Indonesia (Nurlaily et al., 2021). As for results of the investigation regarding the multi-dimensional perceived value framework, it is noted that the three antecedents can all significantly predict perceived value. Interestingly, the impact of PEV on perceived value has been shown to be the strongest with an estimation coefficient of 0.231. This finding suggests that people’s emotional feelings and attitudes can play a critical role in their value perceptions. In the case of China, some studies have implied that Chinese people tend to have a strong sense of national identity and patriotism (Callahan, 2006; Modongal, 2016). Individuals prefer to purchase products made by local companies in China and recent studies have suggested that Chinese customers are pivoting away from foreign brands (Ewing et al., 2002; Chan et al., 2009; He and Wang, 2017). Chinese people might generate a sense of belonging and a higher emotional reward upon using the CBDC, which is legally issued and protected by the PBOC and the government, resulting in a higher perceived value and enhanced behavioral intention to use digital currency.

The research findings are also expected to have important implications for developing marketing strategies for the use of digital currency. As the market of digital currency is rapidly growing, it is increasingly crucial to build coherent and effective marketing strategies to enhance public acceptability. For example, according to the results of the study, it is recommended that decision makers could launch online platforms which contain free resources for improving financial knowledge. This could result in an improvement in perceived value and subsequently in intention to use. Meanwhile, people’s openness to innovation could be greatly enhanced within an innovation-driven culture and building such a culture through various propaganda on communication channels (face-to-face, social media, broadcast media, etc.) would somehow have a positive impact on promoting the usage. Moreover, content marketing strategies could emphasize on the convenience of use which would contribute positively to people’s intention to use digital currency.

In addition, there are some policy implications of the research findings. First, it is advised to have certain policies and practices for strengthening public financial education and introducing more application scenarios, such as transportation, leisure, entertainment, medical care, tourism, etc. This would help enhance people’s financial literacy and usage experience. Second, the government needs to have strict rules and regulations in place to prevent the risk of financial fraud and other financial crimes in the name of digital currency, and great importance should be attached to loss protection, data security and privacy protection. These types of policy responses could help maintain the credibility of digital currency, improve people’s perceived value and encourage the usage. Third, it is recommended to establish some guiding principles by policy makers regarding the design of a convenient digital currency system with greater emphases on expandability and adaptability. Various payment needs from individuals could be conveniently met with the amelioration of using conditions and the coverage of all possible scenarios. Collaboration with commercial banks and telecom operators would help improve the technical infrastructure required for widespread use. Fourth, it is suggested to have some tax relief and subsidy policies for relevant enterprises to encourage the improvement of infrastructures and promote the technological competitions. The government can also establish some mechanisms for direct and low-cost funding to start-ups. These practices could stimulate technological innovations and facilitate the development of the entire digital currency ecosystem, providing a highly safe and convenient usage environment for the general public.

## Conclusion and Limitations

The empirical study aimed at investigating factors which affect people’s intention to use digital currency using empirical evidence from China. The structural equation modeling approach was used to test the hypothesized relationships between variables. The research findings can be summarized as follows: first, a positive nexus between financial knowledge and intention to use was demonstrated and perceived value was also shown to positively affect intention to use; second, perceived value can be significantly predicted by three antecedent variables, including PMV, PFV, and PEV, and it was found to partially mediate the relationship between financial knowledge and intention to use; third, openness to innovation was a significant positive indicator of behavioral intention to use digital currency; fourth, perceived convenience was shown to have a positive impact on intention to use. Confirmation of the causal relationships proposed in the model may inform the choice of policies and the design of marketing strategies which would ultimately help increase people’s intention to use digital currency.

The study has some limitations that can be considered in order to provide clear research direction for future studies. First, it should be noted that the path analysis assumed linear relationships between constructs. If there were some non-linear effects within the model, the results of the path analysis might be slightly distorted. Second, researchers could examine additional factors that may influence people’s intention to use digital currency and increase the total proposed model variance, as the current factors explain 65% of the total variance. Third, this study was only conducted in a single-country context. Different countries with different environments may have different digital currency functions or systems under development, leading to different results in terms of the relationships between variables. It would be interesting to know any other geographically diverse perspectives.

## Data availability statement

The raw data supporting the conclusions of this article will be made available by the authors, without undue reservation.

## Ethics statement

The studies involving human participants were reviewed and approved by Sanda University Ethics Committee. Written informed consent for participation was not required for this study in accordance with the national legislation and the institutional requirements. Written informed consent was obtained from the individual(s) for the publication of any potentially identifiable images or data included in this article.

## Author contributions

GW research design, methodology, data collection, data analysis, supervision, and writing and editing. JY research design, data collection, and writing. QH methodology, data analysis, and writing and editing. All authors listed have contributed to the work and approved it for publication.

## Appendix

**TABLE A1:**

Constructs	Items
Financial knowledge	FK1. I think that I am familiar with finance related terms and concepts.
	FK2. I consider myself to be financially literate.
	FK3. I consider my knowledge of finance to be sufficient.
	FK4. I can make informed judgments of financial products or services.
	FK5. I have the ability to apply basic knowledge in everyday financial choices.
Perceived value	PV1. I believe that digital currency offers me a good value.
	PV2. I think that digital currency has a good quality.
	PV3. I think that it is advantageous to use digital currency for transactions.
	PV4. I think that the basic functions provided by digital currency are sufficient.
	PV5. I think that using digital currency would broaden my horizon.
Perceived monetary value	PMV1. I think that using digital currency has no cost.
	PMV2. I think that digital currency will not lose value over time.
	PMV3. I think that the exchange rate of digital currency is stable.
	PMV4. I think that there are side benefits of using digital currency.
	PMV5. I think that digital currency is a form of wealth.
Perceived functional value	PFV1. I believe that digital currency is better than other mobile payment tools.
	PFV2. I think that digital currency can be used in many scenarios.
	PFV3. I think that transaction settlement via digital currency is safe and fast.
	PFV4. I think that digital currency can help prevent loss of money.
	PFV5. I think that digital currency has good functionality.
Perceived emotional value	PEV1. I think that using digital currency is happy and pleasant.
	PEV2. I think that using digital currency is fulfilling.
	PEV3. I feel content when I use digital currency.
	PEV4. I feel delighted about the implementation of digital currency.
	PEV5. I think that I am not fearful of using digital currency
Openness to innovation	OI1. I think that I am not against innovations.
	OI2. I think that I am interested in innovative technologies.
	OI3. I think that I am open-minded to try new things.
	OI4. I think that I have the courage to envision new possibilities.
	OI5. I think that I am receptive to new information.
Perceived convenience	PC1. I think that there is no difficulty in settling payments via digital currency.
	PC2. I think that digital currency is more flexible to use than paper currency.
	PC3. I think that it is easy to become skillful in using digital currency.
	PC4. I think that it is convenient to use digital currency.
	PC5. I think that using digital currency does not require a lot physical effort.
Intention to use	IU1. I will use digital currency if given the opportunity to do so.
	IU2. I will use digital currency on a regular basis in the future.
	IU3. I have no objection to use digital currency for transactions.
	IU4. I intend to use digital currency frequently for payments.
	IU5. I would recommend my friends to use digital currency.
